# A Comparison Controlled Study Examining Outcome for Children With Autism Receiving Intensive Behavioral Intervention (IBI)

**DOI:** 10.1177/01454455231165934

**Published:** 2023-04-13

**Authors:** Marta Wójcik, Svein Eikeseth, Fillip Ferreira Eikeseth, Ewa Budzinska, Anna Budzinska

**Affiliations:** 1 Oslo Metropolitan University, Norway; 2 Psychiatric Center Ballerup, Mental Health Services, Capital Region of Denmark, Denmark; 3 The Institute for Child Development, Gdańsk, Poland

**Keywords:** intensive behavioral intervention, eclectic intervention, special education outcome, autism

## Abstract

This study evaluated the effects of a center-based Intensive Behavioral Intervention (IBI) model for preschool aged children with autism. Outcomes of 25 children receiving IBI was compared to the outcomes of 14 children receiving autism specific, eclectic, special education. Both provisions were described as appropriate treatment options by the professional agency who diagnosed the children, and the decision of where to enroll the child was made by the parents after consultations with the specialists. After 14 months of treatment, children from the IBI group improved significantly on standard scores in intellectual functioning and adaptive behavior and had a significant reduction in autism severity compared to the children in the autism specific, eclectic, special education group. Results suggest that preschool aged children with autism may make large gains in intellectual and adaptive functioning and improvement in autism severity with IBI, and that effects of IBI may be similar to that of EIBI. These findings must be interpreted with caution due to the limitations inherent in the present comparison-controlled design.

Over the past four decades or so, the number of children diagnosed with autism spectrum disorder (ASD) has risen and demands for effective interventions has increased accordingly. Perhaps the best known comprehensive psychoeducational intervention is Early Intensive Behavioral Intervention (EIBI; Lovaas, 1993, 2003). EIBI employs principles and procedures from Applied Behavior Analysis (ABA; Cooper et al., 2020) to facilitate the acquisition of target behaviors within the areas of communication, play, and social skills and social interest, and to reduce aberrant behaviors and stereotypies and ritualistic behaviors. In EIBI, target behaviors and the order of which these are introduced in the child’s treatment program is informed by developmental psychology, and is tailored to meet the child’s idiosyncratic leaning style and developmental profile. EIBI has been evaluated in numerous outcome studies and meta-analyses, and most of these studies suggest that EIBI may be effective in increasing intellectual and adaptive functioning in many preschool-aged children with ASD compared to eclectic treatment and treatment as usual (e.g., Cohen et al., 2006; Eikeseth et al., 2002, 2007, 2012; Eldevik et al., 2009, 2010, 2020; Howard et al., 2005, 2014; Perry et al., 2009; Reichow et al., 2018; Remington et al., 2007; Rodgers et al., 2021; Sallows & Graupner, 2005; Sandbank et al., 2020; Smith et al., 2000; Waters et al., 2020).

Another ABA-based comprehensive intervention is the Intensive Behavioral Intervention (IBI) model developed by Krantz and McClannahan originating at the Princeton Child Development Institute (McClannahan & Krantz, 1993, 1997, 2001). Treatment begins in a treatment center for children with ASD, and the children are gradually transferred to mainstream kindergartens or schools once they can function and learn effectively from this developmentally integrated setting. A comprehensive staff-training and consumer evaluation system is employed where all staff members including therapists, supervisors and directors receive annual professional evaluations as well as evaluations by the children’s parents (McClannahan & Krantz, 1993, 2001). Strong emphasis is placed on the development of children’s independence by using activity schedules and script and script-fading procedures (Banda et al., 2009; Budzińska et al., 2014; Krantz & McClannahan, 1993; Krantz et al., 1993; Krantz & McClannahan, 1998; Loveland & Tunali, 1991; G. S. MacDuff et al., 1993; J. L. MacDuff et al., 2007; McClannahan & Krantz, 2005; Stevenson et al., 2000).

Like EIBI, IBI utilizes a number of well-researched ABA principles and procedures, most of which are evidence based. Examples of evidence-based principles and procedures used in the IBI is reinforcement, antecedent-based interventions, task analysis, scripts and script fading procedures, response interruption/redirection, prompting, modeling, extinction, discrete trial teaching, video modeling, functional behavior assessment, and functional communication training (Wong et al., 2015).

In contrast to EIBI, there are no studies evaluating global outcomes for children with ASD receiving IBI compared to eclectic special education or treatment as usual. This type of evaluation is important for at least two reasons. First, parents have the need to be informed about the effectiveness and efficacy of interventions offered. Second, this type of scientific knowledge is required by governmental agencies, policy makers and insurance companies so they can make informed decisions about which type of intervention they should endorse.

The present study was designed in an attempt to fill this gap in the scientific literature. We examined whether IBI was more effective than an eclectic autism specific intervention for preschool aged children with ASD. The eclectic autism specific intervention consisted of several different interventions considered by the professional field as being best practice. Children were assessed at intake on intellectual functioning, adaptive functioning, and autism severity and subsequently reevaluated with the same measures after receiving 14 months of intervention. Since EIBI has been found to be more effective than eclectic treatment and treatment as usual, and since IBI has many overlaps with EIBI, we hypothesized that IBI would lead to greater gains in intellectual functioning, adaptive behaviors, and reduced severity of autism compared to the eclectic autism specific intervention.

## Method

### Participants

Participants were 27 children receiving IBI and 29 children receiving eclectic autism specific intervention. All participants in both groups met the following eligibility criteria: (a) an independent diagnosis of autism based on ICD-10 criteria (World Health Organization, 2008); (b) intake chronological age of 5 years or less; and (c) absence of significant medical conditions that could interfere with treatment, such as uncontrollable epilepsy. The diagnosis was set by a multidisciplinary team of independent specialists (psychologist, special educator, and child psychiatrist). The Institute for Child Development (IWRD), Gdańsk, Poland, provided treatment for all children in the IBI group, and all participants who met the eligibility criteria and started at IWRD between 2012 and 2018 entered the experimental group. Participants from the comparison group entered the study between 2014 and 2018 and attended preschools offering services for children with autism in Gdansk and surrounding areas. Written consent was obtained from all participants’ parents or caregivers.

Of the 27 children who entered the IBI group, 25 participated through to the end of the study. Two participants left because they moved out of the area or changed the kindergarten they attended. Of the 29 children who entered the comparison group, 11 participants left because they moved out of the area or changed the kindergarten they attended. Parents of another four children, all from the comparison group, withdrew their consent to participate. This left a total of 14 children in the eclectic autism specific intervention group. Table 1 summarizes the children’s age at intake, gender, parents’ marital status, intake cognitive and adaptive functioning, and autism severity.

**Table 1.** Age, Gender, Intellectual Disability, and Parents’ Marital Status of the Participants in Each Group.

	IBI		Eclectic	
	*M*	*SD*	*M*	*SD*
Age	40,08	6,94	43,93	10,66
Characteristic	IBI		Eclectic	
	*n*	%	*n*	%
Gender				
Male	22	88	12	85.7
Female	3	12	2	14.3
Level of intellectual disability				
None	4	16	4	28.5
Mild	9	36	4	28.5
Moderate	8	32	6	43
Severe	4	16	0	0
Profound	0	0	0	0
Vineland Adaptive Behavior Level				
High	0	0	0	0
Moderately High	0	0	0	0
Adequate	0	0	0	0
Moderately Low	3	12	7	50
Low	22	88	7	50
CARS-2				
Minimal-To-No Symptoms of ASD	0	0	0	0
Mild-To-Moderate Symptoms of ASD	1	4	8	57
Severe Symptoms of ASD	24	96	6	43
Parents’ marital status				
Married	24	96	12	85.7
Not married, divorced	1	4	0	0
Unknown	0	0	2	14.3

*Note*. IBI = Intensive Behavioral Intervention; CARS-2 = Childhood Autism Rating Scales-2.

### Experimental Design

A quasi-experimental design was employed, where participants were assigned to either the IBI group or to the eclectic autism specific group based on parental preferences. Both the IBI provision and the eclectic autism specific provision were described as viable treatment options by the multidisciplinary specialist team (psychologist, special educator, and child psychiatrist) who diagnosed the children. Both provisions were available to all children and based on the information obtained the parents decided whether to enroll the child in the IBI program or the eclectic autism specific program.

### Treatment

#### Intensive Behavioral Intervention (IBI)

The treatment center providing the IBI provision (IWRD) was a certified replication cite for the Princeton Child Development Institute (PCDI), USA, where the IBI model originated from. All personnel from the IWRD were evaluated once a year, based on a PCDI professional evaluation system (McClannahan & Krantz, 1993, 1997). The evaluation lasted approximately for 6 hr, and was carried out as follows: Initially, the evaluator observed the evaluee’s treatment skills, specifically collecting data on on-task behaviors, opportunities to respond (therapist providing the child with learning tasks), behavior-specific praise, correct teaching of new skills, correct teaching of social competence, building relationship with the child, implementing incidental teaching, programing generalization, and decreasing problem behaviors. The evaluator also assessed professionalism and the evaluee’s understanding of ABA principles and procedures. Evaluation of whether the child’s program was individualized and appropriate was assessed by reviewing the child’s program logbook, examining appropriateness of target behaviors, and examining the appropriateness of the procedures used for data collection.

For all behaviors assessed, a 7-point Likert scale was used. Obtaining a mean score of 5.5 or higher was a requirement for employment at IWRD.

The treatment was directed by the fifth author, who was a doctoral level psychologist trained at PCDI. All other treatment personnel held a master’s degree in psychology or pedagogy. The intervention was delivered by a treatment team consisting of four to five persons, including a supervisor, a lead therapist, and therapists. The supervisor had a minimum of 5 years’ experience with the IBI model and had a mean score of 6.8 or higher on the professional evaluation system. The supervisor was responsible for six to eight children and provided approximately 5 hr per week of supervision for each child. Supervision included selection weekly treatment goals, hands-on supervision of staff, and demonstration of treatment procedures. The lead therapist had at least 2 years’ experience working with children with ASD and had earned a mean score of 6 points on the professional evaluation system. The lead therapist was responsible for updating the child’s logbook, making/finding teaching materials, collecting data, analyzing data, and conducting therapy.

Classrooms were divided into three separate areas: (a) a learning area where individual treatment with therapist took place, (b) a leisure skills area, where independent leisure and play skills were targeted, and (c) a play area for break activities.

Each child received 22.5 hr per week of one-to-one treatment. Gradually, the child-therapist-ratio was reduced to one-to-two and one-to-three, depending on the child’s progress and treatment targets. In addition, all children participated in weekly (30-min) external gymnastics and/or music classes conducted by teachers not working at IWRD, with four to seven children in each group. Each child also participated in weekly one-to-one speech-therapy sessions (15–30 min) conducted by a professional speech-therapist.

A number of ABA principles and procedures were used such as differential reinforcement, prompting and prompt-fading, shaping, earless teaching procedure, chaining, and tasks analysis (Cooper et al., 2020), discrete trial training (DTT: Eikeseth et al., 2014), incidental teaching (Hart & Risley, 1975), activity schedules (McClannahan & Krantz, 2010), scripts and script-fading procedures (McClannahan & Krantz, 2005), video modeling (Charlop-Christy et al., 2000).

Programming was based on the PCDI curriculum but was adjusted to meet the specific needs of each child. Each child’s program was individualized and covered all important areas of the student’s life, such as imitation of movements, vocal imitation, matching, receptive and expressive language, social skills and play skills, self-help skills, academic skills, alternative communication, leisure skills, reading, physical education and art. Initial treatment targets focused on establishing a motivation system, and foundational repertoires such as initiating requests, following leisure activity schedules, attending, imitation, matching, following spoken directions, and receptive and expressive labeling. Subsequently, the treatment focused on more advanced cognitive, social, self-care, academic and communication skills. The treatment was delivered in multiple settings, such as separate teaching room, corridors, gym, regular kindergarten, and community settings. The distribution of treatment hours across targets and settings was guided from direct observation and measurement of target behaviors.

On average, the participants had 63 goals on their individualized educational programs. Data were taken at pretest, during acquisition and for generalization. Every 3 months, inter observer agreement (IOA) data were collected for each program.

The treatment was extended to the home settings where parents or caregivers participated in the therapy. The home program was prepared by the lead therapist after consultation with a supervisor. Parents collected data from the treatment twice a month. The data were plotted by the lead therapist. Before parents started the home program, they received training during preschool visits which took place once a week. During preschool visits, the parents were introduced to their child’s educational program, received training on how to implement behavior analytic procedures, and learned how to collect data. The initial parent training focused mainly on maintenance and generalization of acquired skills, then the program was transferred to home settings, subsequently parents started to teach new skills. Once a month the therapist visited the children’s home. At parents’ request the therapist visited more often. On average, participants had 17 goals on their individualized home programs.

A kindergarten for typically developing children was attached to the IBI center, and the participants were transferred gradually to this kindergarten together with their therapist once they had developed prerequisite skills and were able to learn effectively from this developmentally integrated setting.

#### Eclectic Autism Specific Intervention

Children from the comparison group received autism specific eclectic intervention in kindergartens which offered services for children with ASD. A combination of interventions was delivered to best meet the child’s educational needs. The intervention was implemented in a one-to-one format in a separate room by a therapist who was assigned to the child. The treatment included elements from various types of interventions for ASD such as sensory integration therapy (Ayres, 1979), TEACCH (Mesibov et al., 2004; Schopler, Reichler, Bashford, et al., 1995), music therapy (Janzen & Thaut, 2018), the Picture Exchange Communication System (Bondy & Frost, 2001), alternative augmentative communication (Iacono et al., 2016; Schlosser & Wendt, 2008), applied behavior analysis (Cooper et al., 2020), speech and language therapy (Vitásková & Kytnarová, 2017), as well as other methods derived from personal clinical experience. Children also participated in other activities common to preschool programs for typically developing children in their daily routine.

Program goals were individually selected for each child based on the recommendation of parents, teachers, and the director from the kindergarten. Based on those recommendations the teacher prepared the child’s individual educational program. Once a year, the director of the kindergarten evaluated each child’s program.

The program was a full-time program. The children received intervention in the kindergarten, as well as part-time speech therapy and occupational therapy in centers specializing in autism. All specialists working with the child were familiar with the child’s educational program. In addition, therapists provided parent training and one-to-one treatment in the child’s home. Parents were trained to work on social skills and adaptive behaviors.

### Outcome Measures

All children were assessed at intake, and again with the same measures after 14 months of treatment. In all cases, intake assessment was conducted within 2 months of starting the intervention. Intake assessment was carried out in order of referral, and follow-up assessment was carried out in order that children completed 14 months of treatment. Intake and follow-up assessment were carried out by the first and fourth author, who had a master’s degree in psychology and extensive experience with assessing children with ASD.

#### Intellectual Functioning

Intellectual functioning was assessed using the PEP-R (Psychoeducational Profile Revised; Schopler et al., 1990; Schopler, Reichler, Bashford, et al., 1995). The PEP-R assesses the developmental level of young children with autism, who may be non-verbal, have limited attention skills and poor concentration, and who are not used to a formal testing situation. PEP-R provides information on developmental functioning in seven areas: Imitation, Perception, Fine Motor, Eye-Hand Integration, Cognitive Performance, and Cognitive Verbal. The instrument has satisfactory reliability and validity (Delmolino, 2006; Villa et al., 2010), and has been used previously to assess treatment outcomes (Ozonoff & Cathcart, 1998; Panerai et al., 2002, 2009).

The total raw score for all areas was converted into a developmental age (derived from the PEP-R manual), and a Ratio score was calculated by dividing the child’s developmental age by the child’s chronological age, multiplying by 100.

#### Adaptive Behavior

Adaptive Behavior was assessed using the Survey form of the Vineland Adaptive Behavior Scales-II (VABS-II; Sparrow et al., 2005). The VABS-II is widely used to assess adaptive behavior in persons with ASD (Stolte et al., 2016). It assesses adaptive behaviors in four domains Communication, Daily Living, Socialization, and Motor Skills. Based on the domain scores, an adaptive behavior composite (ABC) score is calculated (mean of 100 and *SD* of 15).

#### Autism Severity

Autism severity was assessed using the Childhood Autism Rating Scale (CARS-2; Schopler et al., 2010). The scale rates the following areas: relation to people, imitation, emotional response, body, object use, adaptation to change, visual response, listening response, taste-smell-touch response and use, fear and nervousness, verbal- and non-verbal communication, activity level, level and consistency of intellectual response and general impressions. The total raw score is used to classify children with either minimal-to-no symptoms of autism, mild-to-moderate symptoms of autism, or severe symptoms of autism. The psychometric properties of CARS include high inter-rater reliability and test-retest reliability, good internal consistency and item-total correlation, and a high concordance with the ICD-10 reference standard diagnosis (Russell et al., 2010).

### Data Analysis

The data was analyzed using SPSS version 25 for Windows. All variables were checked for univariate outliers and normality prior to the analyses. Independent Samples *t*-tests revealed that the intervention and control groups differed in pre-treatment adaptive behavior composite score (*p* = .002) and CARS-2 (*p* = .04), but not in intellectual functioning (*p* = .271). Post-treatment outcomes were therefore compared using one-way ANCOVA adjusting for intake scores. Three separate ANCOVA analyses were conducted, one for post treatment intellectual functioning, adjusting for pre-treatment intellectual functioning; one for post treatment adaptive behavior composite score, adjusted for pre-treatment adaptive behavior composite score; and one for post-treatment CARS-2, adjusted for pre-treatment CARS-2. To account for multiple comparisons in our main analysis, the alpha-level was set at 0.05/3 = 0.017. We did not investigate the VABS subscales due to limited power.

To compare how outcome of IBI compares to outcome of EIBI, we used the reliable change index (Jacobson & Truax, 1991). This establishes with 95% certainty whether observed changes at an individual participant level is meaningful and significant in the sense that it highly unlikely can be accounted for by measurement error and/or sample variance. A benchmark for reliable change for EIBI was calculated by Eldevik et al. (2010), based on individual data from almost 300 children with ASD across 16 outcome studies. Eldevik et al. (2010) found that reliable change for intellectual functioning of >26 points, and that reliable change for adaptive functioning of >20 points, and that 30% of the children made reliable change in intellectual functioning, and 21% in adaptive behavior (Eldevik et al., 2010). To compare the effect of IBI to EIBI, we compared the percentage of children achieving reliable change in intellectual functioning and adaptive behavior in the current study to the benchmark established by Eldevik et al. (2010).

We also used the reliable change index to compare the effect of the two different treatments in the current study, by comparing percentage of children making reliable change in intellectual and adaptive functioning in the IBI group to the percentage of children making reliable on the same measures in the eclectic autism specific treatment group.

Explorative correlation analyses were conducted to investigate potential associations between age and post treatment outcomes in both samples.

## Results

Descriptive statistics are presented in Table 1. Means and standard deviations for pre- and post-intervention scores and pre-post intervention differences for all outcome variables are shown in Table 2.

**Table 2.** Means and Standard Deviations of Pre-Treatment, Post-Treatment and Pre-Post Differences for Both Groups.

	Pre-treatment			Post-treatment		Pre-post differences	
	IBI	Eclectic		IBI	Eclectic	IBI	Eclectic
	*M* (*SD*)	*M* (*SD*)		*M* (*SD*)	*M* (*SD*)	*M* (*SD*)	*M* (*SD*)
Intellectual Functioning	51 (18)	57 (15)		75 (24)	63 (21)	24 (18)	6 (15)
VABS							
Total	61 (8)		70 (7)	72 (12)	69 (10)	11 (9)	-1 (7)
Communication	56 (13)		67 (11)	78 (18)	73 (17)	22 (15)	6 (11)
Daily living	65 (10)		74 (9)	75 (13)	74 (10)	10 (10)	0 (8)
Socialization	59 (5)		66 (8)	64 (9)	65 (10)	5 (7)	-1 (8)
CARS-2	45 (6)		39 (9)	34 (7)	36 (9)	−11 (4)	−3 (5)

*Note*. IBI = Intensive Behavioral Intervention; M = mean; SD = standard deviation; VABS = Vineland Adaptive Behavior Scale; CARS-2 = Childhood Autism Rating Scales-2.

Three *1*-*way* between subjects *ANCOVAs* were calculated to examine the effect of IBI controlling for intake scores. Overall, the IBI group showed significant improvements compared to the eclectic autism specific treatment group across all three outcomes: For intellectual functioning, pre-intervention intellectual functioning (covariate) was significantly related to post-intervention intellectual functioning, F (1, 36) = 36.9, p < .001, partial eta squared = .51. The effect of post-intervention intellectual functioning controlled for pre-intervention intellectual functioning was significant, F (1, 36) = 10.1, p = .003, partial eta squared = .22. For VABS total, pre-intervention VABS was a significant covariate, F (1, 36) = 31.1, p = <.001, partial eta squared = .46. The effect on post-intervention VABS total, adjusted for pre-intervention VABS total, was also significant, F (1, 36) = 12.6, p = .001, partial eta squared = .26. Lastly, for CARS-2, pre-intervention CARS-2 was a significant covariate, F (1, 36) = 63.0, p < .001, partial eta squared = .64. Post-intervention CARS-2 controlled for pre-intervention CARS-2 was significant, F (1, 36) = 18.9, p < .001, partial eta squared = .34

**Figure 1.**
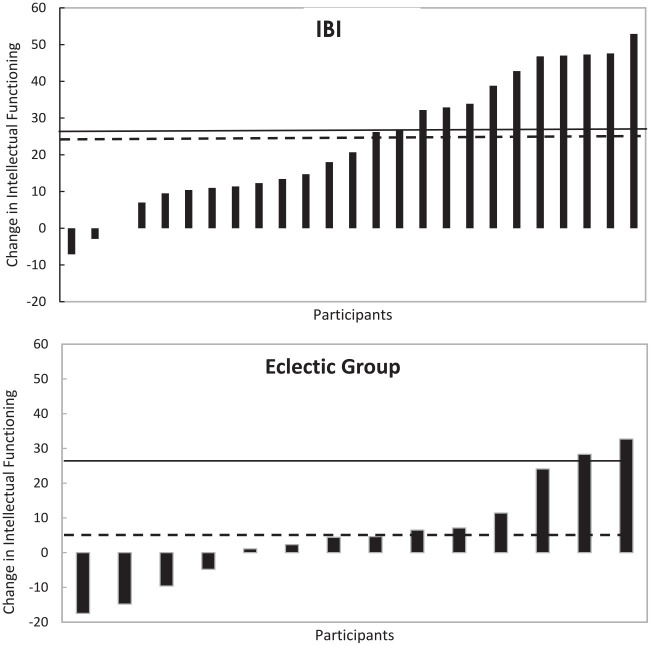
Bars indicate changes in intellectual functioning for IBI (Top) and Eclectic Group (Bottom) for individual children following 14 months of intervention. Results are sorted from highest negative to highest positive. The solid lines represent the Reliable Change benchmarks, >26 for intellectual functioning (Eldevik et al., 2010), the dotted lines represent the mean change in each group in the current study.

Changes in intellectual functioning for the individual children is displayed in Figure 1. As can be seen, 40% (10 of 24 children) of the children in the IBI group met the criterion for reliable change in intellectual functioning (i.e., >27 points change), compared to 14% (2 of 14 children) of the children in the eclectic group. For EIBI, 30% of the children have been found to make reliable change (Eldevik et al., 2010).

**Figure 2.**
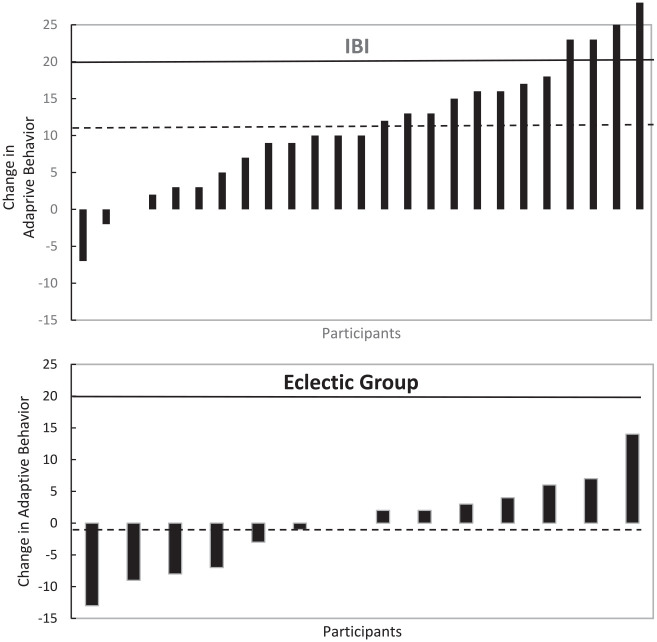
Bars indicate changes in Adaptive Behavior for IBI (Top) and Eclectic Group (Bottom) for individual children following 14 months of intervention. Results are sorted from highest negative to highest positive. The solid lines represent the Reliable Change benchmarks, >20 for Adaptive Behavior (Eldevik et al., 2010), the dotted lines represent the mean change in each group in the current study.

Changes in adaptive behavior for the individual children is displayed in Figure 2. The solid line represents the benchmark for reliable change (Eldevik et al., 2010) and the dotted line represents the mean change of the IBI participants. As can be seen, 16% (4 of 25 children) in the IBI group met the criterion for reliable change in adaptive behaviors (i.e., >20 points change), compared to 0 of 14 children in the eclectic autism specific treatment group. For EIBI, 21% of the children have been found to make reliable change (Eldevik et al., 2010).

As can be seen in Table 3, age was not associated with any outcomes in the IBI group, but had a strong negative association with CARS-2 in the eclectic group.

**Table 3.** Pearson Correlations Between Age at Intake and Post Intervention Outcome Measures.

	Intellectual functioning	VABS total	CARS-2
IBI group	−.327	−.395	−.011
Eclectic group	.323	.280	−.622*

*Note*. IBI = Intensive Behavioral Intervention; VABS = Vineland Adaptive Behavior Scale; CARS-2 = Childhood Autism Rating Scales.

* p < .05.

## Discussion

This comparison-controlled study compared the outcome of the Intensive Behavioral Intervention (IBI) model developed by McClannahan and Krantz (1993, 1997, 2001), to the outcome of an eclectic autism specific intervention. Participants were 39 preschool-aged children with ASD, assessed at intake and after 14 months of intervention on intellectual functioning, adaptive functioning, and autism severity. The children who received IBI made significantly larger gains in intellectual functioning and adaptive behaviors and had a significant reduction in autism severity compared to the children receiving the eclectic autism specific intervention. Chronological age at intake was not associated with outcomes in the IBI group but was negatively associated with autism severity in the eclectic autism specific treatment group.

Using the reliable change index to evaluate outcome (Jacobson & Truax, 1991; see Data Analysis above for an explanation), 40% of the children in the IBI group showed a pre-post gain of 27 points or more on intellectual functioning, hence meeting the criterion for reliable change (Eldevik et al., 2010). In the eclectic autism specific intervention group, only 14% of the children met the criterion for reliable change. For adaptive functioning, 16% of the children in the IBI group met the criterion for reliable change (i.e., gain of 21 points or more; Eldevik et al., 2010), compared to 0% of the children in the eclectic autism specific intervention group. Hence, a higher proportion of participants in the IBI group showed reliable change compared to the participants in the eclectic autism specific intervention group.

In the IPD meta analysis examining outcome of EIBI, Eldevik et al. (2010) found that 30% of the children made reliable change in cognitive functioning and 21% made reliable change in adaptive functioning. Compared to EIBI, IBI seems to produce somewhat lager gains in intellectual functioning and somewhat smaller gain in adaptive functioning. These findings, however, must be interpreted with caution. Firstly, the intervention period of the present study was shorter than the mean intervention period for EIBI (14 months vs. approximately 20 months). A 6 months shorter intervention time may explain why the IBI produced more limited gains in adaptive functioning compared to EIBI. Secondly, cognitive functioning was assessed with different measures. The current study employed the PEP-R (Psychoeducational Profile Revised; Schopler et al., 1990, 1995) whereas EIBI studies used either the Bayley Scales of Infant Development (Bayley, 1969) or the Wechsler Preschool and Primary Scale of Intelligence-Revised (WIPPSI-R; Wechsler, 1989). To our knowledge, no studies have examined the reliability between the PEP-R, Bayley, and WIPPSI-R. Hence, the outcomes of these studies cannot be directly compared. Warranting further replication of our findings

Notably, this is the first comparison-controlled study evaluating outcome of the IBI model. The results add to the current literature showing that IBI can be effective for many preschool aged children with ASD and provides further evidence for the notion that ABA-based interventions may be effective for this client group.

Logically, intervention should start as early as possible after the child has been diagnosed because of the potential for neural plasticity is presumed to be greater the younger the children are (Kolb et al., 2017). Also, younger children have had less time to acquire behavioral delays, and hence may have not fallen so far behind typical development as compared to children that are older. Also, younger children may have had less time to develop and sustain behavioral excesses such as aggression, stereotyped and ritualistic behaviors. In the current study, we assessed weather age at intake was associated with outcome scores. Results showed that this was not the case for either cognitive functioning, adaptive functioning, or for autism severity in the IBI group. This supports findings from some previous studies examining outcome of EIBI (Eikeseth et al., 2002, 2007, 2012; Eldevik et al., 2010). Other studies, however, have found a relation between chronological age at intake and outcome. Smith et al. (2015) found that age at intake predicted outcome on several measures including cognitive functioning, adaptive behavior, and autism severity. Also, Harris and Handleman (2000) found that young age at start of treatment predicted better outcome, when outcome was measured by school placement. Using number of mastered behavioral objectives as an outcome measure, Granpeesheh et al. (2009) found that children between 2-and-5-years of age had better skill acquisition as compared to older children. Two studies found age at intake to be associated with better cognitive outcomes for kindergarten and preschool aged children, but less so in older, school aged children (Blacklock et al., 2014; Perry et al., 2011). This suggests that age of intake may play a greater role for interventions for infants and younger children, as compared to children approaching school age. This possibility merits further study.

The finding that higher intake age was associated with lower autism severity post intervention in the eclectic group was surprising. One may speculate that the younger participants could have been less subject to demands, expectations and structured teaching compared to the older participants. When a child is approaching school age, it may be more pressing to work on targets such as, sitting independently, following social routines, interacting with other children and adults, and to work on language and preacademic skills. If so, this could have resulted in more gains for the older children, a possibility which merits further research. Another possible explanation is that more severe autism typically is detected earlier, potentially leading to a tendency for the older children of having less severe autism characteristics which in turn typically is related to greater intervention gains. That said, if the latter possibility was true, one would expect age to be negatively associated with autism severity at intake, which was not the case (*p* = .256). Moreover, it is unlikely to see gains in autism characteristics independent of gains in intellectual functioning or adaptive behaviors were no significant associations emerged. In sum, the observation of a negative association between age at intake and lower autism severity in the eclectic group was unexpected and possibly a false positive finding. It is in contrast abovementioned research in supporting early intervention for better prognosis (e.g., Smith et al., 2015), and occurred without expected gains in other associated domains.

Predictor values of other intake variables was also assessed, and results showed that intake cognitive functioning was associated with outcome cognitive functioning, outcome adaptive functioning as well as outcome autism severity. The same was the case for intake adaptive behavior and intake autism severity. This is consistent with previous research (e.g., Eldevik et al., 2010; Smith et al., 2015). Interestingly, and also consistent with previous research (e.g., Eldevik et al., 2010), neither intake cognitive functioning, intake adaptive functioning nor autism severity did predict change in scores between intake and follow-up. Taken together, children who scored better at intake, tended to score better at outcome, but they did not make more gains in scores compared to children who scored lower at intake.

Although the study has several strengths, such as certification of IBI personnel, assessment of treatment fidelity, and that IBI conducted at a certified replication site, the study also contained several limitations. Firstly, group assignment was not random, but based on parental preferences. In the current study, parents had an opportunity to enroll their child in either provision, both of which were described as appropriate treatment options by the professional agency who diagnosed the children. The decision of where to enroll the child was made by the parents after consultations with the specialists. Likely, the parents choose the provision they believed to be the best option for their child, and this may have limited potential bias from lack of randomization. Yet, RCT is the criterion standard in scientific intervention studies, hence, there is a need to replicate the current study using RCT. However, both RCT and naturalistic study designs have their own advantages and disadvantages, and also, they address different domains of intended applications. Second, even though the assessors were highly trained and experienced psychologists specializing in ASD, they were not blind or independent of the study. The use of assessors which are not blind to the purpose of the study is a limitation that potentially could have affected the results of the study. Thirdly, a substantial drop-out rate was observed in the eclectic autism specific intervention group; fifteen participants had left the study by the end of the intervention. In contrast, only to two children in the IBI group left the study. The fact that the IBI group had a considerably higher rate of intervention completion than the eclectic autism specific intervention group might be explained, in part, by the significant differences in intervention gains across the two groups in favor of the IBI group. Lastly, the sample size was relatively low, warranting further replication of our findings in higher powered studies.

In sum, this is the first comparison-controlled study evaluating outcome of the IBI model developed by McClannahan and Krantz (1993, 1997, 2001). Results showed that children who received IBI made significantly larger gains in intellectual functioning and adaptive behaviors and had a significant reduction in autism severity compared to the children receiving the eclectic autism specific provision. Children who scored higher at intake, tended to score higher at outcome, but they did not make more gains in scores compared to children who scored lower at intake. Intake age was not associated with outcome. Comparing IBI to EIBI, outcome of IBI seems comparable to that of EIBI, suggesting that IBI can be an effective intervention for preschool aged children with ASD and provides further evidence for the notion that ABA based interventions may be effective for this client group.
